# Contraceptive Use Patterns and Associated Determinants in a Rural Population of Narayanganj District, Bangladesh

**DOI:** 10.1002/puh2.70104

**Published:** 2025-08-09

**Authors:** Maliha Malbika Mimu, S. M. Zobair Hossain, Jheelam Biswas, Mafruha Mahbub, Sabrina Zabin

**Affiliations:** ^1^ Shaheed Suhrawardy Medical College Hospital Dhaka Bangladesh; ^2^ Upazila Health Complex, Bagmara Rajshahi Bangladesh; ^3^ National Institute of Cancer Research and Hospital Dhaka Bangladesh; ^4^ Sher‐E‐Bangla Medical College Hospital Barishal Bangladesh; ^5^ Dhaka Medical College Dhaka Bangladesh

**Keywords:** Bangladesh, contraception, rural community

## Abstract

**Background:**

Contraceptive use patterns in rural communities are shaped by a complex interplay of social and economic factors. In Bangladesh, there is a significant disparity in birth control use between rural and urban areas. This study aims to explore the patterns, practices, and barriers to contraceptive use in a rural community, with the goal of bridging gaps and promoting sustainable development.

**Methods:**

This study combined quantitative and qualitative methods to explore contraceptive use in a rural Bangladesh community. It involved a cross‐sectional survey to assess patterns and perceptions of contraceptive use, followed by focus group discussions (FGDs) to explore practices and barriers within the community.

**Result:**

This study involved 100 married couples from a rural Bangladeshi community, with 69% reporting contraceptive use. Most contraceptive users (81.2%) were women, with oral pills being the most common method. Decisions regarding family size were typically made by husbands (63%). FGDs revealed that husbands are seen as the primary decision‐makers, whereas wives are responsible for contraception. Barriers included lack of sex education, fear of side effects, and noncooperation from husbands, with some women feeling dependent on their husbands to obtain contraceptives. These findings highlight significant cultural and gendered factors affecting contraceptive use in rural communities.

**Conclusion:**

This study highlights how social, economic, and demographic factors shape contraceptive use in rural Bangladesh. These challenges stress the need for gender‐inclusive, culturally tailored interventions to improve contraceptive use and empower rural communities.

## 1 Background

Contraceptive use is a well‐established, cost‐effective public health strategy that offers immediate and long‐term benefits for individuals, families, and communities [1]. Although global progress in family planning has been significant—particularly in high‐income countries where over 90% of users rely on modern methods—many low‐ and middle‐income countries, including Bangladesh, continue to face challenges in achieving equitable access. The national contraceptive prevalence rate (CPR) in Bangladesh stands at 62%, close to the global average of 64%, yet it drops to 59.5% in rural areas [2, 3]. This rural–urban disparity contributes to continued high fertility and population growth in underserved regions [4].

Contraceptive behavior is shaped by a complex interplay of sociodemographic, cultural, and structural factors. Factors such as age, parity, education, income, place of residence, access to healthcare facilities, media exposure, religious beliefs, and spousal communication influence both the likelihood of contraceptive use and the choice of method [5, 6, 7, 8, 9].

To better understand contraceptive behavior, our study draws on the Health Belief Model (HBM) as a guiding conceptual framework. The HBM posits that individual decision‐making around health behaviors—including contraceptive use—is influenced by perceived susceptibility to unintended pregnancy, perceived severity of its consequences, perceived benefits of contraception, perceived barriers to use, and self‐efficacy in adopting a method [9]. Prior studies support these constructs: for example, a Canadian study found that knowledge of contraceptive options and their side effects significantly influenced women's preferences and usage patterns, with higher levels of education associated with greater awareness and a broader range of choices. Similarly, in the United States, men have been found to prioritize contraceptive effectiveness more than women [10]. In contrast, in Bangladesh, contraceptive use is predominantly female‐driven. Male‐controlled or traditional methods remain less prevalent, often due to lack of awareness and social stigma surrounding male involvement in family planning [3]. Research from Nigeria and South Wales further underscores the role of perceived barriers, such as religious and cultural beliefs, fear of side effects, disapproval from partners, and misconceptions about fertility and modern contraceptives [9, 11].

In rural communities, these individual perceptions are further compounded by structural barriers such as limited access to healthcare, socioeconomic dependency, and deeply rooted gender norms that often place the burden of family planning solely on women [12]. These overlapping challenges hinder the effectiveness of national family planning initiatives and contribute to continued disparities in contraceptive use. However, much of the existing research on the Bangladeshi population disproportionately focuses on urban areas, leaving rural contexts underexplored. This study seeks to examine the patterns and determinants of contraceptive use in a rural community of Narayanganj District, Bangladesh. By understanding these challenges in local context, the research aims to generate insights that can inform culturally sensitive and evidence‐based interventions to improve reproductive health outcomes in underserved rural areas.

## 2 Methods

### 2.1 Study Design

This study included both quantitative and qualitative components. The two parts of the study were as follows:

- A cross‐sectional design to assess patterns and practice of contraceptive use using a self‐designed structured questionnaire.
- Two focus group discussions (FGDs) with participants from the same study setting to explore perceptions and barriers related to contraceptive use in their community.

### 2.2 Sample Criteria

Married couples (married for at least 1 month) attending the Upazila Health Complex in Bandar Upazila, Narayanganj, were included in the study. Couples with female spouses who had reached menopause or individuals living separately from their spouses were excluded.

### 2.3 Sample Size

Given the current contraceptive use rate in Bangladesh of 62%, we calculated the sample size using the prevalence formula [2]. With 80% power and a 5% margin of error, the required sample size is 100 married couples.

### 2.4 Data Collection Procedure

Data collection for this study was conducted between January 16 and 18, 2025.

### 2.5 Quantitative Data

Quantitative data were collected using a structured questionnaire designed to assess key domains, including sociodemographic characteristics, contraceptive knowledge and use, access to family planning services, partner communication, and perceived barriers and facilitators to contraceptive use. The questionnaire was pilot tested with 10 married women from a neighboring village to evaluate its clarity, cultural appropriateness, and length. On the basis of the pilot feedback, minor revisions were made to improve the wording and structure.

Subsequently, a total of 100 married couples from the study area were recruited using purposive sampling, in accordance with predefined inclusion and exclusion criteria. The finalized questionnaire was administered face‐to‐face by trained interviewers in Bangla. Each interview lasted approximately 20–30 min.

### 2.6 Qualitative Data

On the basis of survey responses, participants were categorized into contraceptive users and nonusers. From each group, a subset of participants was randomly selected and invited to participate in FGDs.

A total of four FGDs were conducted—two with contraceptive users and two with nonusers. Each FGD included 5–7 couples, with a total of 15 couples participating in the user groups and 10 couples in the nonuser groups. Each session lasted approximately 60–75 min. Discussions were held in a private, community‐based venue to ensure participant comfort and confidentiality. Each FGD was facilitated by a trained moderator fluent in Bangla and familiar with local cultural norms, with assistance from a notetaker. A semi‐structured FGD guide was developed on the basis of themes emerging from the survey findings. It covered domains such as perceived benefits and barriers to contraceptive use, partner dynamics, and social and religious influences. All discussions were audio‐recorded with participant consent, then transcribed verbatim in Bangla and translated into English for analysis by two independent researchers (M. M. and J. B.).

### 2.7 Data Analysis

Quantitative data were analyzed using SPSS version 26.0. Categorical variables were summarized using frequencies and percentages. The association between contraceptive use and sociodemographic variables was assessed using chi‐square and Fisher's exact tests. Means were calculated with a 95% confidence interval, and *p* values <0.05 were considered significant.

Qualitative data were analyzed using a thematic approach, integrating both inductive coding (to identify emerging themes) and deductive coding based on predefined constructs of the HBM. Manual coding w as conducted independently by two researchers (S. M. Z. H.  and M.M.M.) to ensure analytical rigor. Discrepancies in coding were discussed and resolved through consensus to enhance credibility and consistency. Additionally, relevant survey comments were examined in parallel with the coded transcripts to provide contextual depth, without altering the established codes or thematic structure.

### 2.8 Ethical Considerations

Approval for this study was   obtained from ( Letter no: 05.05.0000.003.01.098.24.31 Date: 13/01/2025) Bangladesh Institute of Administration and Management (BIAM) Foundation, Dhaka. All participants were informed of the study's objectives, procedures, and their rights, including the right to withdraw at any time. Written informed consent was obtained prior to participation in both the survey and FGDs. Anonymity and confidentiality were strictly maintained throughout the study process.

## 3 Results

### 3.1 Survey Result

Among the respondents, 44% of couples were aged between 25 and 34 years, and 92% of women reported financial dependency on their male partners. Additionally, 58% of the couples had one or two children (Table 1).

**TABLE 1:** Sociodemographic characteristics of the participants (*n* = 100).

Variables	Frequency (*n*)	Percentage
**Average age of the couples (years)**		
17–24	32	32
25–34	44	44
≥35	24	24
**Educational status of female partner**		
Illiterate	15	15
Primary	33	33
Secondary to higher secondary	45	45
Graduate or higher	7	7
**Educational status of the male partner**		
Illiterate	6	6
Primary	32	32
Secondary to higher secondary	54	54
Graduate to postgraduate	8	8
**Economic status**		
Poor	17	17
Lower middle class	52	52
Middle class	28	28
Upper middle class	3	3
**Economic dependency**		
Wife dependent on male partner	92	92
Both partners economically independent	8	8
**Number of living children**		
No child	16	16
1–2	58	58
>3	26	26

Overall, 69% of couples were actively using contraceptives, with 81.2% of these cases involving use by the wife alone. Notably, more than half (53.6%) of contraceptive users reported irregular use. The most commonly used method was the oral contraceptive pill (52.2%), followed by implants (17.4%) and male condoms (13.2%). Decisions regarding family size were predominantly made by husbands (63%) or in‐laws (9%), whereas only 23% involved joint or wife‐led decision‐making. Furthermore, 73% of contraceptive users reported needing their spouse's permission to use contraception (Table 2).

**TABLE 2:** Pattern and practice regarding using contraceptives (*n* = 100).

Variables	Frequency (*n*)	Percentage
**Use of contraceptives**		
Yes	69	69
No	31	31
**Who uses contraceptive (*n* = 69)**		
Both	2	2.8
Husband	11	15.9
Wife	56	81.2
**Types of contraception method used (*n* = 69)**		
Permanent	8	11.6
Temporary	61	88.4
**Type of contraceptive used (*n* = 69)**		
Condom	9	13.2
Intrauterine devices	2	2.8
Implant	12	17.4
Ligation	7	10.1
Oral pill	36	52.2
Natural method	3	4.3
**Frequency of contraceptive use (*n *= 69)**		
Irregularly	37	53.6
Regularly	32	46.3
**Getting permission from spouse**		
No	27	27
Yes	73	73
**Decision about family size**		
Social and religious rules	5	5
Husband's decision	63	63
Decision of in‐laws	9	9
Wife's decision	23	23

Significant associations between contraceptive use and variables such as the age of the couple, number of living children, the spouse using contraception, and the decision‐maker regarding family size were observed. Couples aged 25–34 years and those with one to two children were more likely to use contraceptives than other groups. Contraceptive use was significantly higher among female partners; however, use was strongly influenced by the husband's authority over family planning decisions (Table 3).

**TABLE 3:** Factors associated with contraceptive use (*n* = 100).

	Use of contraceptives		*p* value
	Frequency, *n* (%)		
Variables	Yes	No	
**Average age of the couples**			
17–24	15 (21.7)	17 (54.8)	**0.005^c^**
25–34	35 (50.7)	9 (29.0)	
≥35	19 (27.5)	5 (16.1)	
**Number of living children**			
No child	6 (8.7)	10 (32.3)	**0.012^c^**
1–2	43 (62.3)	15 (48.4)	
≥3	20 (29.0)	6 (19.4)	
**Education of female partner**			0.416^c^
Illiterate	13 (18.8)	2 (6.5)	
Primary	22 (31.9)	11 (35.5)	
Secondary to higher secondary	30 (43.5)	15 (48.4)	
Graduate or higher	4 (5.8)	3 (9.7)	
**Education of male partner**			
Illiterate	5 (7.2)	1 (3.2)	0.735^f^
Primary	23 (33.3)	9 (29.0)	
Secondary to higher secondary	35 (50.7)	19 (61.3)	
Graduate or higher	6 (8.7)	2 (6.5)	
**Economic dependency**			
Wife dependent on male partner	65 (94.2)	27 (87.1)	0.249^f^
Both partners economically independent	4 (5.8)	4 (12.9)	
**Which spouse uses contraceptives**			
Husband	11 (15.9)	0	**0** **.001^f^**
Wife	56 (81.2)	2 (6.5)	
Both	2 (2.9)	0	
None	0	29 (93.5)	
**Decision about family size**			
Social and religious rules	2 (2.9)	3 (9.7)	**0.001^f^**
Husband's decision	44 (63.8)	19 (61.3)	
Wife's decision	20 (29.0)	3 (9.7)	
Decision of in‐laws	3 (4.3)	6 (19.4)	

*Note:* c = chi‐square test done; f = Fisher exact test done; *p* value <0.01 considered significant.  Bold values reffer to significant p values.

### 3.2 Interview Result

Regarding the practice of contraceptives, two major themes were identified.

### 3.3 Husband's Role in Decision‐Making

Ten out of 15 couples agreed that the husband should decide on the type of contraceptives used, as he is typically seen as the breadwinner and head of the household.

One 29‐year‐old woman shared: “My husband runs this family. He takes all the decisions. So he will decide what I shall do, including birth control.” (R5).

### 3.4 Wife's Responsibility for Contraception

All 15 couples agreed that the wife should be responsible for contraception. Two couples followed practices from other female family members, 10 felt ashamed to obtain condoms from female healthcare workers, and 2 believed it was natural for the wife to manage birth control.

One 34‐year‐old husband explained: “Because women bear the children, it is determined by God that she should take necessary steps for birth control.” (R15).

Regarding barriers to contraceptive use, three major themes were identified.

### 3.5 Ignorance

Two out of 10 couples admitted that they did not receive any sex education before marriage and were unsure about which contraception methods were suitable for them. They also felt ashamed discussing contraception.

An 18‐year‐old newlywed couple shared: “Nobody told us what to do before marriage. It is shameful to speak about such things outside the bedroom.” (R4).

### 3.6 Fear of Invasive Methods and Side Effects

Five out of 10 couples expressed fear about contraception, particularly invasive methods like injections or intrauterine devices. They also worried about side effects like menstrual irregularity and reduced sexual pleasure with barrier methods.

A 19‐year‐old husband said: “My wife fears injections. We've heard oral pills cause issues at our age, and I don't want to use condoms because they create a barrier between us.” (R7).

### 3.7 Noncooperation From Husband

Three women expressed frustration with their husbands’ lack of cooperation. As they were fully dependent on their partners, they relied on them to obtain contraceptives. However, their husbands often gave excuses, such as the high cost of contraception, or ignored the issue entirely.

A 40‐year‐old woman shared: “My husband never takes responsibility for our children. He also doesn't do anything to prevent more children from coming.” (R1).

## 4 Discussion

In the current study, 69% of participants reported using contraceptives, which is significantly higher than the contraceptive use rate in rural Bangladesh and above the national CPR [2, 3]. The majority (88.4%) of participants used temporary methods like oral pills and implants, similar to trends in Bangladesh and low‐ and middle‐income countries like Ethiopia and Nigeria [3, 9, 13]. Even in high‐income countries like the United States and Canada, short‐term methods are preferred [10, 14]. In contrast, young women in neighboring countries like India often favor permanent methods due to financial compensation and limited social independence [15]. This preference for temporary methods in the current study may be driven by cultural norms, availability, and the convenience and reversibility of these methods.

The findings of the current study reveal a significant gender imbalance in contraceptive responsibility, with 81.2% of users being women. This scenario is prevalent throughout Bangladesh, where male involvement in family planning remains significantly low in both urban and rural areas [16]. Interviews with our participants revealed that husbands, as the primary breadwinners, typically make decisions about contraception, whereas women are solely responsible for adopting birth control methods, regardless of their preferences. Contraceptive use is often tied to the husband's authority in determining family size. A systematic review supports this, showing that in patriarchal societies, women predominantly use contraception, but men often influence the decision [17]. This scenario is similar to countries like India, Cambodia, Pakistan, Afghanistan, and Nepal, where women's status is tied to their childbearing ability, and they have limited autonomy in healthcare decisions due to economic dependence on husbands and in‐laws [18, 19, 20].

Age and number of children were key factors in contraceptive use in our study. Participants aged 25–34 with 1–2 children had the highest use, reflecting a trend in Bangladesh where women in their peak reproductive years prioritize family planning [21]. This pattern is similar to trends observed in other Asian countries, such as Malaysia and Pakistan, where couples over the age of 25 are more likely to use contraceptives compared to younger couples. Contraceptive use tends to increase with the age of the couples [20]. Younger couples (17–24 years) were less likely to use contraception, with interviews revealing that lack of premarriage sex education led to misconceptions and fear. This mirrors findings from African countries like Nigeria and South Ethiopia, and Asian countries like Vietnam, Nepal, and India where barriers such as lack of sex education, misconception about fertile period, shyness, rumors, family pressure, and cultural norms prevent contraceptive use [9, 20, 22]. In contrast, sex education was unrelated to contraceptive use in developed countries [23].

Other barriers to contraceptive use in our study included fear of invasive methods and lack of cooperation from husbands. These findings are similar to those in African and Southeast Asian countries, where fear of side effects and partner disapproval are significant barriers [20, 24, 25]. Rural women in India and ethnic minority women in Korea often perceive contraception as undesirable, untrustworthy, socially unacceptable, and frequently unnecessary [20, 25]. Studies showed that increasing male involvement in family planning increases contraceptive use as well as maternal and child health [26]. In Cambodia, Nepal, Pakistan, and India, a positive attitude from husbands and the involvement of mothers‐in‐law in family planning have been associated with increased birth intervals and greater use of modern contraceptive methods [20].

Irregular use of contraceptives in our study (53.6%) underscores persistent barriers to sustained adoption. Although we could not find the exact reason behind this pattern, a study in rural Kenya found that irregular use of contraception is linked to stockouts, misconceptions about side effects, and inadequate counseling by healthcare providers [27]. Addressing these barriers requires community‐based interventions that engage both men and women, alongside healthcare providers, to normalize contraceptive use.

## 5 Limitations

Our study has several limitations. First, it was conducted at a single center with a small sample size, limiting the generalizability of the findings. Additionally, the study did not provide in‐depth exploration of other important factors such as economic status, access to health care facilities, and cultural and religious influences on contraceptive use among the study population.

## 6 Conclusion

This study highlights the influence of social, economic, and demographic factors on contraceptive use in rural Bangladesh. Although adoption rates are moderate, persistent barriers such as gendered decision‐making, cultural misconceptions, and economic dependency continue to hinder consistent use. However, more research is needed to understand the underlying causes of irregular contraceptive use, which was reported in this study. Future research involving larger and more diverse populations is recommended to validate and expand on these findings.

## Author Contributions

**Maliha Malbika Mimu**: conception and design, collection and assembly of data, manuscript writing and revision. **S. M. Zobair Hossain**: collection and assembly of data, manuscript writing and revision. **Jheelam Biswas**: conception and design, collection and assembly of data, data analysis and interpretation, manuscript writing and revision. **Mafruha Mahbub**: collection and assembly of data, data analysis and interpretation, manuscript writing and revision. **Sabrina Zabin**: manuscript writing and revision.

## Conflicts of Interest

The authors declare no conflicts of interest.

## Transparency Statement

The lead author Dr. Maliha Malbika Mimu affirms that this manuscript is an honest, accurate, and transparent account of the study being reported; that no important aspects of the study have been omitted; and that any discrepancies from the study as planned have been explained.

## Data Availability

All data relevant to the study are available in Mendeley Data; doi: 10.17632/n32j46zw95.1.
